# The Knockdown of *AUXIN RESPONSE FACTOR 2* Confers Enhanced Tolerance to Salt and Drought Stresses in Tomato (*Solanum lycopersicum* L.)

**DOI:** 10.3390/plants12152804

**Published:** 2023-07-28

**Authors:** Ibtihaj El Mamoun, Sarah Bouzroud, Mohamed Zouine, Abdelaziz Smouni

**Affiliations:** 1 Laboratoire de Biotechnologie et de Physiologie Végétales, Center of Plant and Microbial Biotechnology, Biodiversity and Environment, Faculty of Sciences, Mohammed V University in Rabat, Rabat 10000, Morocco; ibtihaj_elmamoun@um5.ac.ma; 2 Laboratoire de Recherche en Sciences Végétales, UMR5546, Université de Toulouse, Centre National de la Recherche Scientifique (CNRS), Université Toulouse Paul Sabatier (UPS), Toulouse-INP, 31320 Auzeville-Tolosane, France; 3 Microbiology and Molecular Biology Team, Center of Plant and Microbial Biotechnology, Biodiversity and Environment, Faculty of Sciences, Mohammed V University in Rabat, Rabat 10000, Morocco; s.bouzroud@um5r.ac.ma

**Keywords:** *SlARF2*, auxin, salinity, drought stress, tolerance, tomato

## Abstract

Auxin response factors (*ARFs*) act as key elements of the auxin-signaling pathway and play important roles in the process of a plant’s growth, development, and response to environmental conditions. We studied the implication of the *SlARF2* gene in the tomato response to salt (150 mM of NaCl) and drought (15% PEG 20000) stresses. The functional characterization of *SlARF2* knockdown tomato mutants revealed that the downregulation of this gene enhanced primary root length and root branching and reduced plant wilting. At the physiological level, the *arf2* mutant line displayed higher chlorophyll, soluble sugars, proline, and relative water contents as well as lower stomatal conductance and a decreased malondialdehyde content. Moreover, *SlARF2* knockdown tomato mutants demonstrated higher activities of the antioxidant enzymes superoxide dismutase (SOD) and catalase (CAT) under salt and drought stresses than the wild type. Indeed, the stress tolerance of the *arf2* mutant was also reflected by the upregulation of stress-related genes involved in ROS scavenging and plant defense, including *SOD*, *CAT*, *dehydration-responsive element-binding protein*, and *early responsive to dehydration*, which can ultimately result in a better resistance to salt and drought stresses. Furthermore, the transcriptional levels of the *Δ1-pyrroline-5-carboxylate synthase* (*P5CS*) gene were upregulated in the *arf2* mutant after stress, in correlation with the higher levels of proline. Taken together, our findings reveal that *SlARF2* is implicated in salt and drought tolerance in tomato and provides some considerable elements for improving the abiotic stress tolerance and increasing the crop yields of tomato.

## 1. Introduction

Drought and salt are the most common abiotic stresses, adversely disturbing plant growth and productivity [1]. Plant responses to abiotic stresses are tremendously complex and rely on the activation of multiple signaling pathways in order to minimize damages while preserving valuable resources for growth, development, and reproduction [2]. Plant hormones such as abscisic acid (ABA), ethylene, and salicylic acid (SA) play a pivotal role in the set of plant responses to stresses [2]. The plant’s auxin, indole-3-acetic acid (IAA), which is the key regulator of many aspects of plant growth and development, was recently proposed as a key player in plant responses to environmental stresses [3,4,5]. Auxin action occurs through the transcriptional regulation of auxin response genes, which is primarily mediated by the following three types of transcriptional regulators: auxin response factors (ARFs), the short-lived nuclear protein Aux/IAA, and TOPLESS (TPL) [6,7]. ARFs modulate auxin action by interacting with auxin-responsive elements (AuxRE) located in the promoter region of auxin-responsive genes, thereby regulating their transcription and plant growth and metabolism [8]. 

The ARF gene family was identified and well-characterized in several plant species such as Arabidopsis (*Arabidopsis thaliana*), rice (*Oryza sativa*), sorgho (*Sorghum bicolor*), banana (*Musa acuminata*), popular (*Populus trichocarpa*), physic nut (*Jatropha curcas*), Chinese cabbage (*Brassica rapa*), soybean (*Glycine max*), and maize (*Zea mays*) [9,10,11,12,13,14,15]. In tomato (*Solanum lycopersium*), 22 ARFs were previously isolated and well characterized by Zouine et al. (2013). Co-transfection assays identified five ARFs as activators and eight as repressors [7].

The auxin response factor gene family is involved in the control of many physiological processes including embryogenesis, leaf expansion and senescence, lateral root development, and fruit set and development [16,17,18,19,20,21,22]. A functional analysis revealed the involvement of *ARF3* in the lateral root development of *Arabidopsis* [16,23]. In tomato, this transcriptional regulator controls epidermal cells and trichome formation [24]. Tomato *ARF4* plays an important role in cotyledon development and hypocotyl growth and negatively regulates chlorophyll accumulation and starch synthesis in fruits [17,18], while *SlARF5*, *SlARF7,* and *SlARF8* act as regulators of fruit set and parthenocarpy [20,25,26]. *ARF*s are also involved in plant responses to environmental stresses. Expression profiling revealed the responsiveness of *ARF* genes to a wide range of abiotic stresses including salt and water deficit in many plant species such as sorghum (*Sorghum bicolor*), banana (*Musa acuminata* L.), soybean (*Glycine max*), hot pepper (*Capsicum annuum*), peanut (*Arachis hypogaea* L.), and oil palm (*Elaeis guineensis* Jacq.) [27,28,29,30]. In tomato, the expression levels of many *SlARF* genes were altered in response to abiotic stresses, namely salt and water deficit [31,32].

*ARF2* has been extensively studied for its role in the regulation of several plant developmental processes including leaf senescence, floral abscission, seed size and weight, and fruit development and ripening [22,33,34,35,36]. Identified as a repressor, its overexpression in tomato leads to a blotchy ripening phenotype, resulting from the significant accumulation of ripening-related genes and metabolites [36]. Meanwhile, *SlARF2* knockdown affects root development, leading to an enhanced root branching [35], which is an important trait observed in salt-tolerant genotypes [37]. Moreover, emerging evidence previously suggested *SlARF2* involvement in plant responses to abiotic stresses [31]. However, no studies have focused on the functional characterization of *ARF2* in stress conditions. Therefore, within this study, morphological, biochemical, physiological, and molecular analyses were conducted to assess the function of *SlARF2* in tomato response to salt and drought stresses. 

## 2. Results

### 2.1. SlARF2 Gene Displays a Strong Expression in Different Tomato Organs 

**Figure 1:**
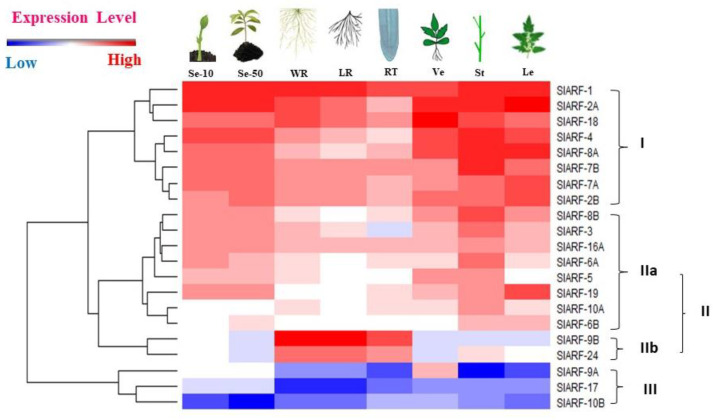
Heatmap of the expression levels of tomato *ARF* genes in different vegetative tissues. The distance used is dependent on Euclidean distance, which allows for the clustering of gene expression by expression levels. The expression value corresponds to the mean of normalized expressions of all tomato cultivars contained in the TomExpress platform (according to RNA-Seq data). Genes highly or faintly expressed in the tissues are colored red and blue, respectively. Se-10, seedlings (10 days); Se-50, seedlings (50 days); WR, whole root; LR, lateral roots; RT, root tips; Ve, Vegetative (35 days); St, stems; and Le, leaves, as schematically represented above the displayed array data.

To address the expression pattern of the *SlARF*2 gene in different vegetative tissues, we monitored the mRNA level of *ARF*s of all tomato cultivars present in the online TomExpress platform (according to RNA-Seq data) [38]. Following the heatmap data, *SlARF*s can be categorized into three distinct groups based on their expression profiles. The *SlARF2* gene belongs to the first group that gathered genes exhibiting high expression levels in all tomato vegetative tissues. According to the RNA-seq results, *SlARF2* showed a dynamic expression pattern, with a strong expression in the leaves and a low expression in the root tips (Figure 1).

### 2.2. SlARF2 Is Induced by Salt and Drought Stresses

**Figure 2:**
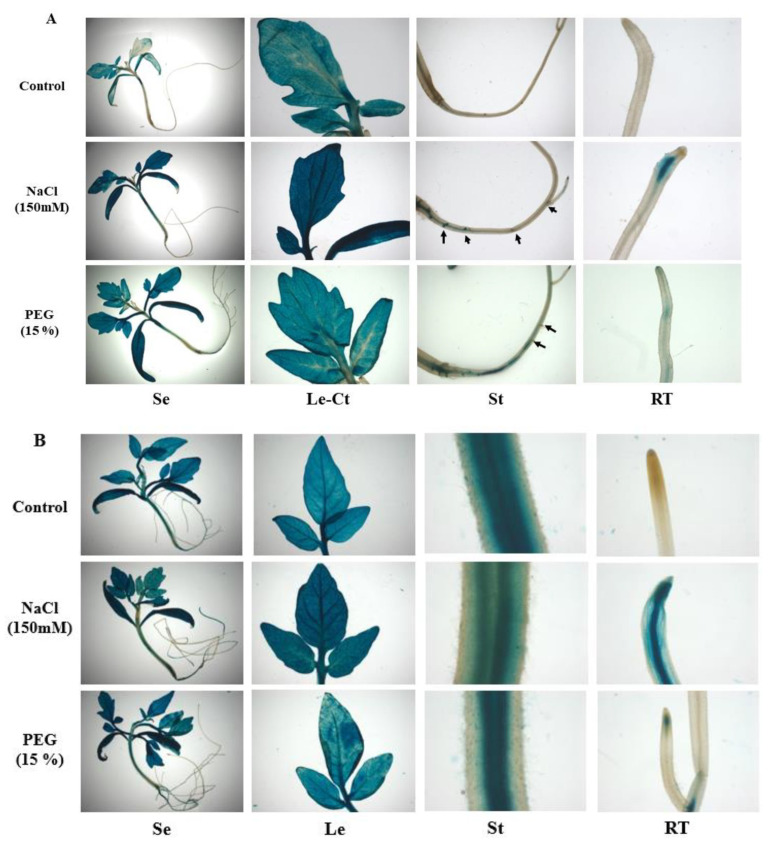
Tissue-specific expression of *SlARF2AB* fused to the GUS reporter gene driven by the *SlARF2* promoter in seedlings after 48 h (**A**) and 5 days (**B**) of salt (NaCl = 150 mM) and drought (PEG 20000 = 15%) stresses. Histochemical staining present in spots, represented by arrows, corresponds to lateral root initiation sites in seedlings treated with salt and PEG after 48 h. The expression pattern was analyzed in 3-week-old Se, seedling; Le, leaves; Ct, cotyledon; St, stem; and RT, root tips. The images are representative of at least three independent experiments with 9 seedlings per experiment.

Two-week-old transformed homozygous pro*SlARF2*::GUS seedlings were treated with 150 mM NaCl or PEG 20000 at 15% for 48 h and 5 d. Our data reveal that *SlARF2* showed a strong pattern of vascular expression in many tissues in response to abiotic stress. The results also show the absence of blue staining in the root tip under the control condition, which is in concordance with the *SlARF2* expression pattern (Figure 1). The GUS histochemical staining assays of the transgenic plants showed, after 48 h of stress, strong GUS signals in all the examined tissues, including in the cotyledon, leaf, and stems. Importantly, *SlARF2* expression was not only limited to vascular tissues, as it was also strongly detected in the root tips and lateral root initiation sites (Figure 2A). The GUS gene expression driven by the *SlARF2* promoter was strongly induced in the root tips (Figure 2B) as well as in the vasculature after 5 d of stress. 

### 2.3. Downregulation of SlARF2AB Improves Growth and Physiological Parameters in Salt and Drought Stress Conditions

**Figure 3:**
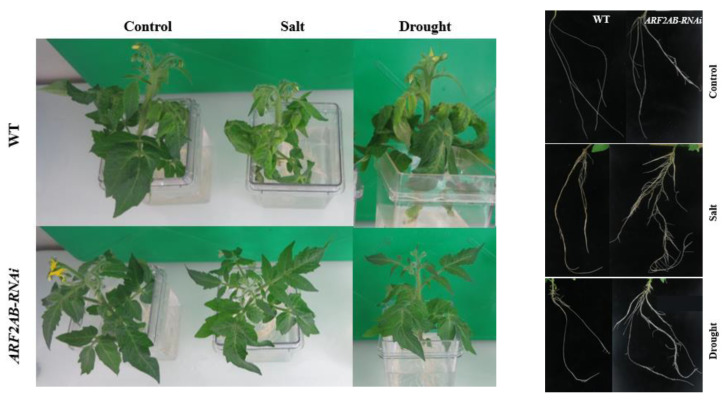
Phenotypic changes in MicroTom (WT, wild type) and transgenic tomato plants *ARF2AB-RNAi* under control, salt (NaCl = 150 Mm), and drought treatments (PEG 15%) for 15 days.

We analyzed the drought and salt tolerance of 6-week-old *ARF2AB-RNAi* and wild-type (WT) tomato plants. Under stress conditions, the WT plants showed more withering and leaf yellowing compared with the *ARF2AB-RNAi* transgenic plants that remained healthy and showed vigorous growth performance. Chlorosis was more prominent in the leaves of the WT, especially at 150 mM of NaCl, whereas the *ARF2AB-*RNAi plants displayed less chlorosis (Figure 3). 

**Figure 4:**
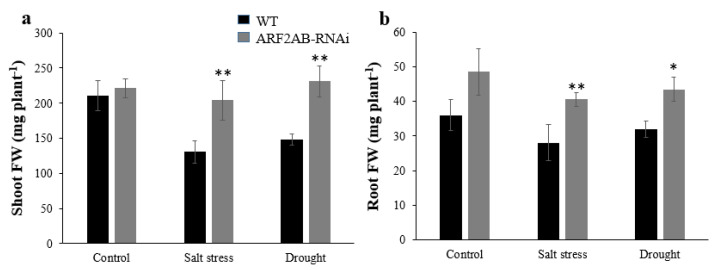

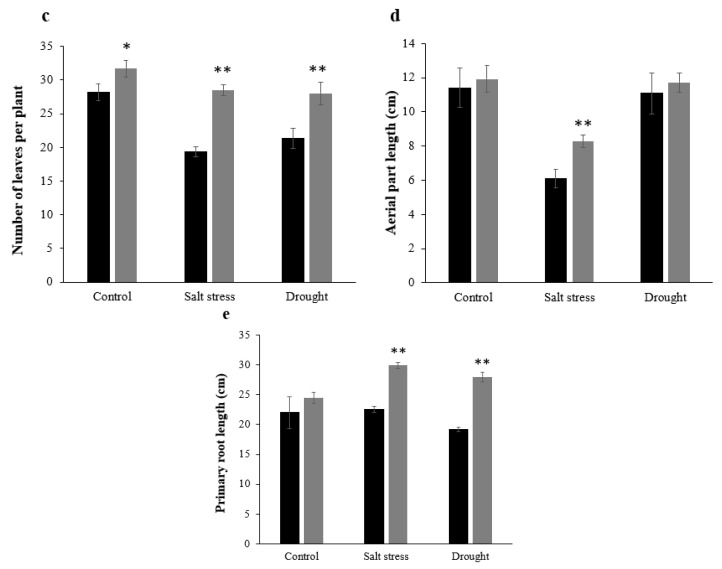
*SlARF2AB-*RNAi and WT plant responses to salt and drought tolerance in tomato. Comparison of shoot (**a**) and root (**b**) fresh weight, number of leaves (**c**), aerial part length (**d**), and primary root length (**e**) of transgenic and wild-type plants under normal and stress conditions. Six-week-old seedlings of transgenic and wild-type plants were grown with 150 mM NaCl or with PEG 20000 at 15% or in the absence of stress (control) for two weeks. Data are means ± SE of three biological replicates. Each replicate sample was a composite from nine seedlings. Asterisks indicate significant differences between transgenic lines and the wild type. * *p* < 0.05; ** *p* < 0.01, Student’s *t* test.

The growth of the WT plants was severely affected by stress compared with the transgenic plants, as judged by the shoot and root weight per plant (Figure 4a,b). In response to salt stress, the fresh weight decreased by 38% and 22% in the leaves and roots of the WT plants, respectively, and only by 7% and 16% in the *ARF2AB*-RNAi plants. Under drought stress, the shoot fresh weight per plant of the *ARF2AB-RNAi* plants increased by 4.4% compared to the normal condition. However, the shoot fresh weight of the WT plants decreased by 30%. Additionally, the number of leaves per plant in the *ARF2AB-RNAi* plants was also higher than those in the WT plants in all the tested conditions (Figure 4c). The plant height was also considerably higher by 35% in the mutant than the corresponding values of the WT after salt treatment (Figure 4d). We noticed a significant increase in the primary root length of the transgenic plants after being treated with salt by 22% and with drought by 14% (Figure 4e), while no significant difference was observed in the primary root length between the unstressed and stressed WT plants. 

**Figure 5:**
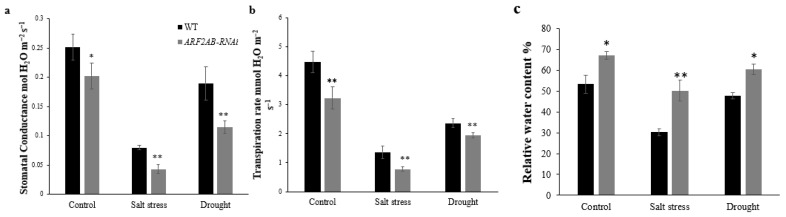
Response of transgenic line and WT plants to drought and salt stresses. Comparison of stomatal conductance (**a**), transpiration rate (**b**), and relative water content (**c**) in leaves of unstressed and stressed plants. Six-week-old seedlings of transgenic lines and the wild type were treated at 150 mM of NaCl (salt stress) and PEG 20000 at 15% (drought stress) for 15 d. Data are means ± SE of three biological replicates with at least nine seedlings for each replicate. Asterisks indicate significant differences between transgenic lines and the wild type. * *p* < 0.05; ** *p* < 0.01, Student’s *t* test.

Investigating the stomatal status under normal and stress conditions revealed a significant difference in the stomatal conductance between the *ARF2AB-RNAi* and wild-type plants. Indeed, the stomatal conductance was significantly higher in the WT plants than in the *ARF2AB-RNAi* mutants. In response to salt or drought stress, the *ARF2AB-RNAi* mutants displayed a significantly lower stomatal conductance than the WT plants (Figure 5a). This decrease reached 78% and 43% after the exposure to salt stress and drought stress, respectively. Similar findings were recorded for the transpiration rate (Figure 5b). Our data show a significant reduction in the transpiration rate in mutants by 75% and 40% in response to salt stress and drought stress, respectively, whereas the decrease was nearly 69% and 47% in the WT plants. Besides the stomatal index, the stress resistance of a plant depends on the evaporating surface area. The transgenic and WT plants exhibited statistically similar fresh weights under normal conditions. However, the *ARF2* transgenic plants possessed markedly higher leaf and root fresh weights than the WT after salt and drought stresses (Figure 4a,b). The RWC was higher in the *ARF2AB-*RNAi plants compared with the WT plants under both stressed and unstressed conditions (Figure 5c). The RWC decreased by 57% after salt stress in the WT plants, whereas in the silenced plants, the decrease was nearly 25%. 

### 2.4. Under-Expression of SlARF2-Enhanced Chlorophyll, Sugars, and Proline Contents in Salt and Drought Stress Conditions

**Figure 6:**
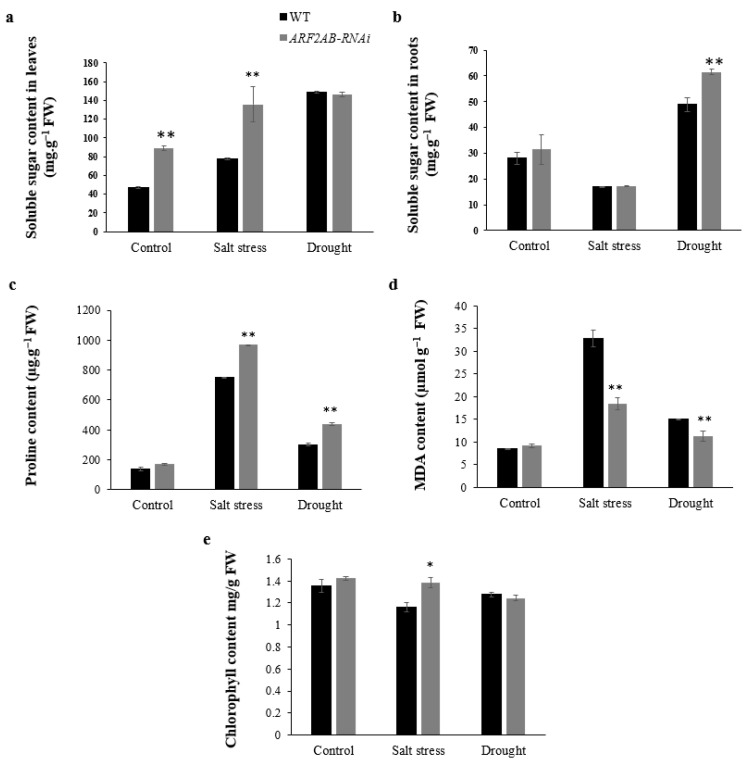
Changes in soluble sugars in leaves (**a**) and roots (**b**); proline (**c**), MDA (**d**), and chlorophyll (**e**) contents in response to salt and drought stresses. Six-week-old seedlings of transgenic lines and the wild type were treated with 150 mM of NaCl and PEG 20000 at 15% for 15 d. Data are means ± SE of three biological replicates with nine seedlings for each replicate. Asterisks indicate significant differences between transgenic lines and the wild type. * *p* < 0.05; ** *p* < 0.01, Student’s *t* test.

We analyzed the changes in the levels of some biochemical markers in the transgenic and WT plants under stress conditions. The results indicate that the chlorophyll content declined in both mutants and in the WT plants under drought stress, while the content was the same under normal conditions. After being exposed to NaCl, the WT plants exhibited a marked decrease (14% reduction). Meanwhile, the Chl content was significantly higher (by 19%) in the *ARF2AB*-RNAi plants compared to the WT, and still maintained the same level observed in the normal conditions (Figure 6e). Under normal growth conditions, soluble sugars were significantly higher in the *SlARF2AB*-RNAi leaves (89 mg.g^−1^ FW) than in the WT (46 mg.g^−1^ FW) (Figure 6a). Under stress, the soluble sugar content increased in the transgenic and WT plants. However, this increase was more pronounced in the *ARF2AB-*RNAi plants subjected to salt stress (52%), while the increase was around 64% for the WT plants compared to the control. The soluble sugar content of the WT plants under drought stress remained the same as that of the mutant. In the roots, the soluble sugar content in the WT plants under drought stress conditions increased (73%), but it remained noticeably lower than that in the transgenic plants (96%) (Figure 6c). At a 150 mM concentration of NaCl, no significant difference was observed in the soluble sugar content of the *ARF2AB-*RNAi and WT seedlings. In the absence of stress, the proline content was the same in the transgenic plants compared to the WT. Under stress, both the WT and mutants showed an increase in the proline content (Figure 6c). The proline amount increased by 28% and 45% in the *ARF2AB*-RNAi plants compared to wild-type plants under saline and drought conditions, respectively. 

### 2.5. SlARF2AB-RNAi Transgenic Plants Displayed Lower MDA with an Increase in Antioxidant Enzyme Activities in Response to Salt and Drought Stresses

Under normal conditions, no significant changes in the MDA content were observed for the *SlARF2AB-RNAi* and WT plants. The MDA content, however, increased in both the WT and transgenic seedlings under stress conditions. This increase was significantly higher in the salt-stressed WT plants than in the *ARF2AB-*RNAi plants (Figure 6d). The MDA content increased by 3.85-fold in the WT, whereas a 2-fold increase was recorded in the mutant. The downregulated line, when exposed to drought, showed a lower MDA content, which was maintained at a level similar to that in the unstressed plants. The WT plants, however, had a higher MDA content under drought treatment (75%).

**Figure 7:**
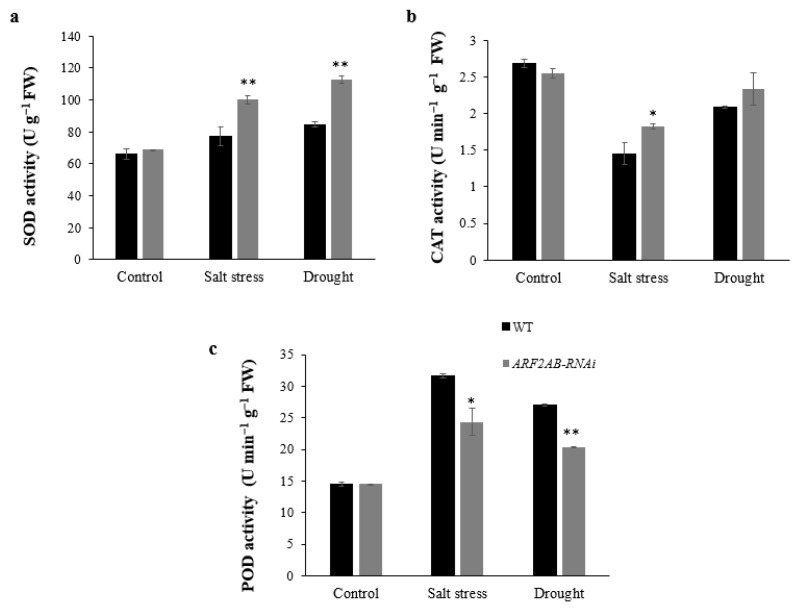
Superoxide dismutase (SOD) (**a**), catalase (CAT) (**b**), and peroxidase (POD) (**c**) activities in leaves of transgenic and wild-type plants under normal and stress conditions. Six-week-old seedlings of transgenic lines and the wild type were treated with 150 mM of NaCl and PEG 20000 at 15% for 2 weeks. Data are means ± SE of three biological replicates with nine seedlings for each replicate. Asterisks indicate significant differences between transgenic lines and the wild type. * *p* < 0.05; ** *p* < 0.01, Student’s *t* test.

Furthermore, the transgenic plants exhibited higher activities of the two antioxidant enzymes superoxide dismutase (SOD) and catalase (CAT) under stress conditions (Figure 7a,b). The SOD activities in the *ARF2AB*-RNAi plants increased by 46% and 63% in response to salt and drought stresses, respectively, whereas the WT plants displayed much lower values and increased by 16% and 27%. However, the peroxidase (POD) activity significantly increased in both the WT and transgenic tomato under stress (Figure 7c). The WT plants showed a greater increase of 118% and 86% in the POD activity in the salt- and drought-stressed leaves, respectively.

### 2.6. Stress-Related Genes Are Regulated by Salt and Drought Stresses in SlARF2 Knockdown Mutant

**Figure 8:**
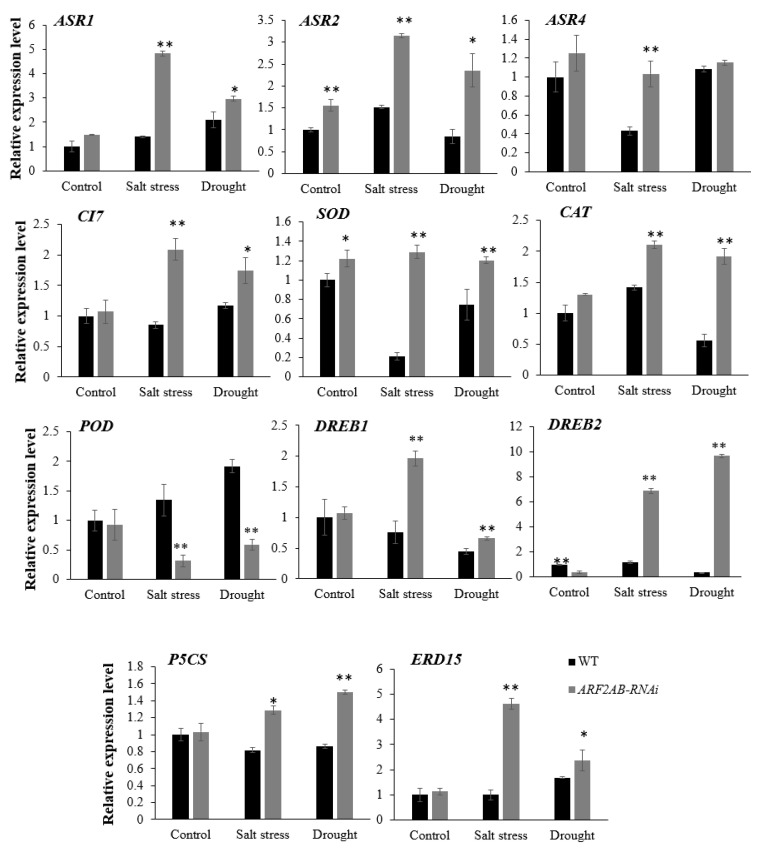
Transcript levels of *ASR1*, *ASR2*, *ASR4*, *CI7*, *SOD*, *CAT*, *POD*, *DREB1*, *DREB2*, *P5CS,* and *ERD15* in leaves were altered in *ARF2AB-*RNAi line in response to salt and drought stresses. Six-week-old seedlings of the transgenic line and wild type were treated with 150 mM of NaCl and PEG 20000 at 15% for 15 d. These seedlings were used to collect samples for RNA extraction. The transcript levels were normalized to *SlActin*. Expression levels of these genes in transgenic plants are indicated as relative to the level of the wild type, which was set to 1, referring to the transcripts of *SlActin* in the same samples. Data shown are means ± SE of three biological replicates with nine seedlings for each replicate. Asterisks indicate significant differences between transgenic line and wild type. * *p* < 0.05; ** *p* < 0.01, Student’s *t* test.

**Figure 9:**
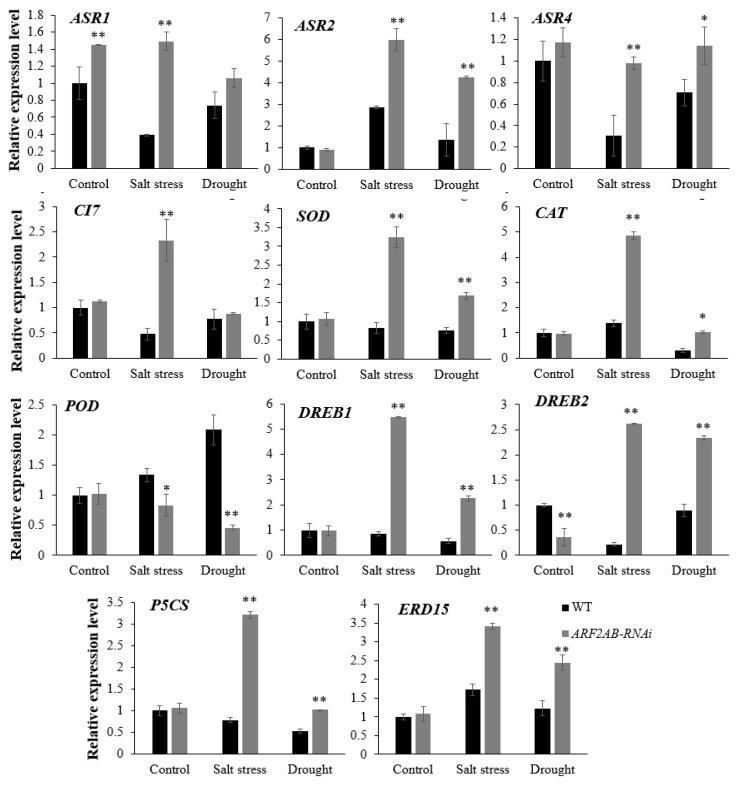
Transcript levels in roots of *ASR1*, *ASR2*, *ASR4*, *CI7*, *SOD*, *CAT*, *POD*, *DREB1*, *DREB2*, *P5CS,* and *ERD15* were altered in *ARF2AB-*RNAi line after salt and drought stresses. Six-week-old seedlings of transgenic line and wild type were treated with 150 mM of NaCl and PEG 20000 at 15% for 15 d. These seedlings were used to collect samples for RNA extraction. The transcript levels were normalized to *SlActin*. Expression levels of these genes in transgenic plants are indicated as relative to the level of the wild type, which was set to 1, referring to the transcripts of *SlActin* in the same samples. Data shown are means ± SE of three biological replicates with nine seedlings for each replicate. Asterisks indicate significant differences between transgenic line and wild type. * *p* < 0.05; ** *p* < 0.01, Student’s *t* test.

The expression of some stress-related genes that are frequently used as biomarkers (ABA stress ripening (*ASR*), cold inducible 7 gene (*CI7*), *SOD*, *CAT*, *POD*, dehydration-responsive element-binding protein (*DREB*), Δ1-pyrroline-5-carboxylate synthase (*P5CS*), early responsive to dehydration (*ERD15*)) was analyzed in the *ARF2AB-*RNAi transgenic line and WT plants growing under normal and stressed conditions via real-time RT–PCR analysis (Figure 8 and Figure 9). The silencing of the *ARF2*-induced expression of stress-related genes was observed both in the leaves and roots.

The expression of the *SlAsr1*, *SlAsr2,* and *SlAsr4* genes appeared to be highly induced in the leaves and roots in the transgenic plants than in the WT plants. A higher upregulation in gene expression was observed in the response to salt stress than to drought stress. The expression level of *SlCI7*, the salt and drought marker, was significantly upregulated in the *SlARF2AB*-RNAi leaves and roots after the exposure to stress, while no significant changes were detected in the WT plants. As shown in Figure 8 and Figure 9, the expression levels of *SOD* and *CAT* in the leaves and roots were greatly upregulated in the transgenic lines that were subjected to salt and drought stresses. Conversely, the expression levels of these genes exhibited lower mRNA levels in the WT plants in comparison to the non-treated plants. Under normal conditions, no significant difference was detected in the expression of *POD* between the WT and *SlARF2AB*-RNAi plants. The results show that the *POD* expression was only downregulated in the transgenic line to a significant level. The transcriptional levels of the *DREB1* and *DREB2* transcription factors were upregulated in both the leaves and roots in the transgenic plants under the two stressed conditions. Moreover, these plants exhibited a higher expression of the target genes compared with the WT. Furthermore, the *SlDREB1* expression was greatly elevated during salt stress, whereas *SlDREB2* was strongly induced by drought stress. Compared with the control, the transcriptional levels of the *P5CS* gene, which encodes a key enzyme in proline biosynthesis, were upregulated in the *ARF2AB-RNAi* plant after stress, which is consistent with the higher levels of proline detected in the *SlARF2AB*-RNAi plants. *ERD15* was identified among the drought-induced genes and was used as a stress-responsive gene [39,40]. The analysis showed a transcript upregulation in the transgenic line by all the stress treatments. These results indicate that *SlARF2AB* downregulation would improve tomato tolerance to salt and drought stresses by modulating the expression of stress-related genes. 

## 3. Discussion

Abiotic stress negatively affects the growth and development of many crop species, including tomato, regarding germination, vegetative growth, flowering, and fruit set and ripening [41]. Most tomato cultivars are relatively sensitive to salt and drought, and thus fail to produce high yields in a fragile environment [42]. Genetic engineering techniques were previously developed to control yield losses due to abiotic stresses, but minimal progress has been made due to the complex mechanism of stress tolerance. Genome editing was applied in tomato improvement, mainly in the context of improving fruit yield and quality [43,44] and stress resistance [45,46,47]. The downregulation of SlUGT75C1 (uridine diphosphate glycosyltransferases) increased ABA and ethylene in the silenced fruits and hastened fruit ripening. The knockdown mutants also exhibited tolerance to drought stress [47]. It was reported that the deletion of the tomato SlAGL6 (AGAMOUS-LIKE6) using CRISPR/Cas9 technology led to the development of parthenocarpy and even showed improved yielding under heat stress without compromising the weight, fruit shape, or pollen vitality [46]. Thus, gene knockdown is used as a plant precision breeding method for crop improvement. However, the genetic improvement of tomato fruit productivity via genome editing in response to abiotic stresses remains relatively unexploited. Auxin is involved in regulating organogenesis and patterning processes occurring during several aspects of plant growth and development [48]. Previous reports revealed that environmental stress signals are integrated into changes in auxin homeostasis and signaling [49,50,51], and ARFs are the main transcription factors in the auxin signaling pathway [52,53]. In rice, it was found that *OsARF11* and *OsARF15* showed differential expression under salt conditions [54]. In support to these results, Du et al. (2013) [55] reported that most *OsARF* genes were responsive to drought stress. Xu et al. (2016) [56] showed that in tea plant, some of the *CsARF* genes were up- or downregulated in the shoots and roots in response to salt and drought stresses and that they may play roles in the crosstalk between the auxin and stress signaling pathways. Also, many *CaARF* genes were regulated by abiotic stresses in pepper [57]. Some *DnARF*s that are involved in abiotic stress tolerance were reported in *Dendrobium officinale* [51]. In *Brachypodium distachyon*, Liu et al. (2018) [58] reported that the BdARF8, BdARF10, and BdARF18 genes were significantly upregulated under salt and PEG treatments. Studies conducted on chickpea revealed that *CaARF4.2* was significantly upregulated under salt treatment [59]. Tang et al. (2018) [13] suggested that *JcARF2* and *12* were upregulated under salt treatment, and *JcARF1* and *16* were induced after drought stress in physic nut. Likewise, in Jerusalem artichoke, under salt stress, the expression of *ARF2* was sharply increased [60]. Kang et al. (2018) [61] showed that a sweet potato *IbARF5* is involved in salt and drought tolerance in transgenic *Arabidopsis*. Furthermore, a set of *EgARF*s were also upregulated under salt and drought stress conditions in oil palm [21]. Recently, in peanut, *ARF18* likely enhanced salt tolerance through the posttranscriptional regulation of miR160 [62]. In tomato, the knockout and knockdown of the *SlARF4* gene enhanced salt and drought stress tolerance [8,32]. The promoter region of the ARF genes harbors a great number of *cis*-acting elements associated with abiotic stress, suggesting that ARFs might be involved in stress tolerance, and a high number of these stress-associated motifs were identified for *SlARF2* [31,58]. These previous studies suggest that *ARF2* can play a central role in plant responses to abiotic stresses. However, further studies should be conducted to confirm the involvement of *SlARF2* in stress response. 

### 3.1. SlARF2 Gene Expression Is Induced by Salt and Drought Stresses

The *SlARF2* mRNA levels contained in the online TomExpress platform showed various accumulations in all plant parts (Figure 1). This result was similar to the expression pattern of *SlARF2A/B* in tomato [35]. According to previous reports, both the *SlARF2A* and *SlARF2B* genes responded to salt and drought stresses, suggesting that they might participate in abiotic stress responses [31]. This observation led us to examine the spatiotemporal expression of pARF2::GUS *in planta*. We found that the *SlARF2* promoter is strongly induced in the root tips and lateral root initiation sites after 48 h and 5 d of stress. In Arabidopsis, *ARF2* was detected in the vascular tissue and in the initiation sites of lateral roots [63]. Meng et al. (2015) [64] reported a strong expression of the ProARF2:GUS construct in the root differentiation zone and in the mature leaf abaxial epidermis. However, Yu et al. (2017) [57] reported that *CaARF2* is highly expressed in cotyledons. In tomato, we previously demonstrated that the expression of *SlARF2A* and *SlARF2B* were significantly regulated by salt and drought stresses [31]. In the present study, we were able to confirm the regulation of the expression of the *SlARF2* gene by salt and drought stresses by analyzing the tissue-specific expression of this gene using *SlARF2-GUS* transgenic plants. 

### 3.2. ARF2AB Silencing Confers Enhanced Salt and Drought Tolerance in Tomato

Physiological indices are characteristic parameters for evaluating plants’ responses to abiotic stresses. Auxin is a key regulator of root development, and the increased root branching might improve plants’ water uptake efficiency [65]. In our study, the morphological and physiological responses of both the wild type and transgenic tomato line grown under unstressed conditions were statistically similar. It is worth recording that better root development of the transgenic tomato is an important factor in increasing biomass and enabling plants to cope with abiotic stresses (Figure 4b,e). Lovelli et al. (2012) [66] demonstrated that higher root growth and biomass accumulation characterized salt tolerance response and low water potential of tomato under stress. Okushima et al. (2005) [67], Okushima et al. (2007) [68], and Narise et al. (2010) [69] showed that the *AtARF7/AtARF19* double mutant is altered in lateral root formation and gravitropism in *Arabidopsis*. Indeed, the overexpression of cherry *CpARF7* promoted root growth and increased lateral roots, which led to the improvement of the drought resistance of tomato plants [70]. Furthermore, Marin et al. (2010) [16] revealed that in the lateral root primordium, the tasiRNAs inhibit *ARF2*, thus promoting lateral root growth. At the same time, studies have revealed that the *ARF2* is a regulator that is involved in negatively controlling ABA-mediated seed germination and primary root growth [71]. In addition, the overexpression of mango *MiARF2* inhibits the root growth of *Arabidopsis* [72]. Effectively, the primary root length of treated SlARF2AB-RNAi-stressed plants was significantly higher than the untreated ones and the WT (Figure 4e), suggesting that the *ARF2* gene expression affects and enhances root branching (Figure 3), as demonstrated previously by Hao et al. (2015) [35]. Also, it was addressed that the overexpression of *ARF2* leads to abnormal root architecture with shorter primary roots in response to low potassium stress [73]. This is in accordance with the study conducted by Hao et al. (2011) [74] and Tiwari et al. (2021) [75], which reported a decreased expression of *ARF2* in transgenic *Arabidopsis* lines under abiotic stress, implying its role in lateral root initiation and development as *ARF2* repressed root growth. Likewise, in alfalfa, the knockout of the *MtARF2* gene increased the lateral root density [76]. Choi et al. (2018) [77] demonstrated that the loss of function of the *arf2* mutants caused longer root hairs to grow. The *ARF2* gene indirectly represses cell cycle genes via the indirect repression of Plethora (PLT) genes, thus maintaining the activity of stem cells and regulating root development [78]. In fact, *ARF7* upregulates the expression of *ARF2*, which, in turn, represses meristematic and patterning genes [79]. Moreover, the leaf senescence of the *atarf2* mutant is delayed [22]. Furthermore, the *AtARF2*, *AtARF*7, and *AtARF*19 genes were induced by senescence, and mutations in *AtARF*7 and *AtARF19* increased the *atarf2* phenotypes [34]. 

The water status and balance between the water supply and transpiration rate under stress conditions was evaluated. The *ARF2AB-RNAi* plants preserved higher relative water contents (RWC) (Figure 5c) and presented higher numbers of leaves (Figure 4c), thus leading to a higher fresh biomass. In addition, the shoot fresh weight was higher in the transgenic plants, most likely because of the better growth of root systems allowing for plants to cope with stress more efficiently. This finding is consistent with a previous study, in which the improved tolerance to drought stress in *arf2* mutants was mostly associated with their capacity to maintain a higher leaf RWC [64]. These phenotypes were also reported for the *arf2/mnt1* mutation, which can cause the increased growth of aerial organs and extra cell proliferation [22]. Indeed, pleiotropic effects of *ARF2* are mediated through the negative regulation of the transcription of developmental genes. In fact, *SlARF2* is a transcriptional repressor, so it is thinkable that the decreased functioning of *SlARF2* may result in less repression of the auxin signaling pathway, leading to an improved tolerance to abiotic stress by altering the plant architecture. In the present study, the decrease in the chlorophyll contents in the RNAi plants was significantly less important compared with the WT plants and was also concomitant with lower oxidative damage, suggesting that the downexpression of *SlARF2AB* in tomato resulted in increased photosynthetic capabilities in stress conditions. Furthermore, the reduced chlorophyll content can also be due to a lower amount of water loss from the leaves. The stomatal conductance and transpiration rate are strongly associated with the leaf osmotic potential and water retention capacity in plants [80]. Meng et al. (2015) [64] demonstrated that the *ARF2* knockdown mutants accumulate ABA, consequently resulting in an increase in stomatal closing, reducing transpiration, which eventually leads to stress tolerance, which causes crosstalk between ABA and auxin. Accordingly, the enhanced tolerance of transgenic tomato can be attributed, at least in part, to their lower transpiration rates. The stomatal conductance is then decreased, which is apparently one of the major factors contributing to the stress tolerance of *SlARF2AB-RNAi*. Previous studies revealed that plant tolerance to abiotic stresses are closely related to physiological responses, which are mostly described by the accumulation of low-molecular-weight metabolites such as soluble sugar and free proline, which are important indicators that are directly involved in the adjustment of osmotic potentials in plant cells [81]. As shown in Figure 6, after salt and drought stress treatments, soluble sugar, and proline, which is a well-known osmolyte, increased more in the transgenic plants, which can effectively alleviate osmotic stress and oxidative damage induced by stress. Malondialdehyde (MDA) is a key marker that is generally used to estimate oxidative lipid injury in response to abiotic stress [82]. In this regard, our physiological measurements indicated that the *SlARF2AB*-RNAi plants notably decreased the MDA contents in response to salt and drought stresses compared to the wild type, suggesting that silencing the *SlARF2AB* gene directly or indirectly leads to beneficial physiological changes involved in osmotic adjustment as well as better cell viability through scavenging redundant ROS. 

### 3.3. SlARF2AB-RNAi Modulates the Expression of Stress-Related Genes in Tomato under Salt and Drought Stress Conditions 

In our study, the *SlARF2AB*-silenced plants revealed a significant induction of several stress-related genes, which is something that is considered to be beneficial in the resistance to abiotic stress [83]. We found that *SlAsr1* showed a higher expression pattern in the leaves after stress in mutants. *Asr* genes were previously reported to be induced by abiotic stress in several plant species including tomato [84]. Indeed, *Asr1* was reported to be upregulated and was shown to confer salt tolerance [85]. In addition, protein interaction assays demonstrated that ARF2A interacts with the ASR1 protein [36]. *SlAsr4* expression seems to be rather weak compared to the other candidate genes. This could explain previous studies [86], in which *SlAsr4* expression could not be detected after 24 h in stressed tomato. *SlAsr2* presented relatively low expression in leaves compared to roots. Maskin et al. (2001) [87] found that *Asr2* transcripts are highly abundant in roots in response to drought stress. The expression of the *CI7* gene, a homolog of *Arabidopsis COR47* and potato *CI7* dehydrin, used as stress markers [88], was upregulated in both leaves and roots, thus validating the efficiency of the abiotic stress treatment in our experiments. 

Plants contain efficient reactive oxygen species (ROS) scavenging pathways involving enzymatic antioxidants, including SOD, POD, and CAT, to protect the plants from oxidative-stress-induced cell damage [89]. In fact, SOD acts as ROS scavenging by converting abundantly available superoxide to H_2_O_2_, while CAT consequently detoxifies H_2_O_2_ into H_2_O, and POD participates in the ROS release or consumption [90,91]. Our results reveal that higher transcripts of these genes were detected in the transgenic plants, suggesting that they might have more efficient antioxidant defense machinery compared to the WT plants. This is consistent with the higher SOD and CAT activities and decreased expression level of the cell wall POD and reduced MDA levels under salt and drought stress treatments (Figure 7). Taken together, these observations reveal that *SlARF2* can be an *ARF* gene with pleiotropic effects in response to abiotic stress in tomato. Several studies revealed the common stress signaling transduction pathways of dependent ABA and independent ABA, which have become models in plant stress [92]. Like *AtDREB2A/B*, the *SlDREB1* belongs to the A-2 subgroup of the AP2/EREBP subfamily and is involved in the adaptation responses to drought stress [93]. In *Arabidopsis*, the *AtDREB2A/B* were two transcriptional activators implicated in dehydration-inducible gene expression through an ABA independent pathway recognizing DRE/CRT [94]. Furthermore, *SlDREB2* was identified as a salt-stress-regulated transcription factor, and its overexpression in tomato and *Arabidopsis* mediates salt stress tolerance by affecting multiple cellular processes [95]. Previous studies have shown that auxin acts as a positive regulator in ABA-sensitive and ABA-dependent tomato and *Arabidopsis* plants [66,96]. In our study, both salt and drought stress induced the expression of the *SlDREB1* and *SlDREB2* genes in the transgenic plants, suggesting that the expression of these genes might play a key regulatory role in the transcriptional activation of stress-induced genes involved in the ABA signal transduction pathway. Previous studies reported that the high level of ABA increases the transcript level of *P5CS* [97]. Meanwhile, *SlP5CS*, a main gene that is involved in proline biosynthesis and is positively associated with proline content, was also detected. The *SlARF2AB*-RNAi plants had higher expression levels of *SlP5CS* under abiotic stress (Figure 8 and Figure 9), thereby explaining the higher proline amounts detected in the transgenic plants (Figure 6c). Accordingly, our results indicate that silencing *SlARF2AB* leads to the upregulation of these genes as a transcriptional regulator or, otherwise, leads to the interaction with other genes to alter the expression of transcripts encoding regulatory proteins that are involved in anti-stress metabolism in tomato. All of these reports revealed that the *ARF2 g*ene has various roles in several hormone signaling pathways and might function as a significant connecting junction in the plant’s response to abiotic stresses. 

## 4. Materials and Methods

### 4.1. Plant Materials

To evaluate the functional significance of *ARF2* and its effect on the physiology of transgenic plants, transformation was performed in tomato (*Solanum lycopersicum*, L. cv Micro-Tom). *SlARF2* is encoded by two genes, *SlARF2A* (Solyc03g118290.2.1) and *SlARF2B* (Solyc12g042070.1.1) [7]; thus, transgenic lines simultaneously that were silenced for both genes were previously generated and well described by Hao et al. (2015) [35]. Confirmed double-knockdown tomato lines suppressed the expression of *SlARF2AB* using RNAi, wild-type tomato (WT), and a reporter line pARF2::GUS were used in this study.

### 4.2. Histochemical Analysis of Gus Expression

To visualize GUS activity, transgenic lines bearing pARF2::GUS were cultivated in square Petri dishes containing 50% Murashige and Skoog medium (MS) and then placed in a growth chamber with 16 h/8 h (light/dark) photoperiod at 25 °C. Seven-day-old seedlings were transferred in tanks containing aerated Broughton and Dillworth (BD) nutrient solution [98]. Salt and drought were applied to three-week-old tomato seedlings, and each treatment was performed by adding 150 mM of NaCl and 15% PEG 20000 for drought stress to the culture tank. After 48 h and 5 days of stress, plants were immersed overnight in GUS staining solution (pH 7.2), 3 mM X-gluc, 0.1% Triton X-100, 50 mM Na_2_HPO_4_/NaH_2_PO_4_, and 10 mM EDTA at 37 °C, and were vacuum pumped and then decolorized using several washes of graded ethanol series. As control, plants were maintained in BD liquid medium.

### 4.3. Plant Growth and Stress Treatment Assays

WT tomato and *SlARF2AB-RNAi* seeds were sterilized for 10 min in 50% sodium hypochlorite, washed 5 times with sterile distilled water, and then sown in square Petri dishes containing half-strength MS medium in a controlled climate room at 25 ± 2 °C with 16 h/8 h (light/dark) photoperiod, 80% relative humidity, and 250 mol m^−2^ s^−1^ intensity light. Three-week-old seedlings were then cultured in BD nutrient solution, after acclimatization, for three more weeks. Six-week-old seedlings were then subjected to control condition and salt (150 mM of NaCl) and drought (15% PEG 20000) stress treatments for 2 weeks. Every 3 days, the hydroponic solution was renewed for each treatment to keep the well growth condition. Each treatment included three biological replicates. Leaf and root tissues collected from the various treated and untreated plants were frozen immediately in liquid nitrogen and stored at −80 °C until analysis. 

### 4.4. Determination of Morphological and Physiological Traits 

To analyze the alterations in plant architecture between WT and *SlARF2AB-RNAi* plants after 2 weeks of treatment, many parameters were measured. Shoot and root fresh weights (FW) of the stressed and unstressed plants were determined, and the mean was obtained from 18 seedlings of three independent experiments. The ImageJ 1.53g software (https://imagej.nih.gov/ij/) was used to measure the number of leaves per plants, total leaf area, aerial part, and primary root length. Three technical replicates were performed for control and stress conditions.

#### 4.4.1. Measurement of Chlorophyll Content 

For chlorophyll (Chl) content, each leaf sample (0.1 g) from stressed and unstressed plants was ground in liquid nitrogen and extracted with 2 mL of acetone/hexane (4:6 *v*/*v*). The extract was centrifuged at 10,000 rpm for 1 min and the absorbance of the supernatants was read at 645 and 663 nm. Total chlorophyll content was calculated using the following formulas according to the method of Wellburn (1994) [99]: Total Chl = 20.29 × A_645_ + 8.02 × A_663_.

#### 4.4.2. Determination of Soluble Sugar Content 

Soluble sugar content was evaluated based on the methods by Riazi et al. (1985) [100] and Jin et al. (2007) [101] using the reagent anthrone method with glucose as the standard. Both roots and leaves were selected for determination. Thus, 100 mg of ground samples were homogenized in 2 mL of 80% ethanol in shaker for 1 h. Extracts were centrifuged at 6000× *g* for 10 min and then transferred into a new test tube, and equal volume of chloroform was added. After centrifugation at 12,000× *g* for 10 min, the aqueous part was mixed with anthrone solution and then incubated in boiling water bath for 15 min, and the cooled samples were read at 620 nm using a spectrophotometer.

#### 4.4.3. Determination of Leaf Stomatal Conductance and Transpiration Rate

Leaf transpiration (E) and stomatal conductance (gs) of fifth fully expanded leaf from the base of stressed and unstressed plants were determined using a portable steady-state porometer LI-1600 (Li-Cor Inc., Lincoln, NE, USA) under the following conditions: temperature 22–25 °C; 16 h/8 h (light/dark) photoperiod; relative humidity 80%; and 250 mol m^−2^ s^−1^ intensity light. The porometer consists of a cuvette with a broadleaf aperture (2 cm^2^), which permits the precise measurements of water loss by transpiration (µg cm^−2^ s^−1^) and stomatal resistance (s cm^−1^). Measurements were carried out by attaching the cuvette to the leaf surfaces and simultaneously registered humidity (%) conditions and temperature (°C). Three biological replicates were conducted at each condition.

#### 4.4.4. Determination of Relative Water Content 

Expanded leaves from each tomato plant were excised and their FW were recorded immediately. The turgid weight (TW) of the excised leaves was recorded after floating them overnight in deionized water at 4 °C. Afterwards, leaves were dried for 2 days at 60 °C and the dry weight (DW) was determined. Relative water content (RWC) was calculated using the following equation: RWC = (FW − DW)/(TW − DW) × 100. 

#### 4.4.5. Determination of Proline Content

Proline content was determined using the method described by Zhang et al. (2009) [102]. Briefly, 200 mg of ground leaf tissue was homogenized in 4 mL of 3% sulphosalicylic acid at 100 °C for 10 min. Subsequent to centrifugation at 12,000× *g* for 2 min, 2 mL supernatant was added to 2 mL acid ninhydrin reagent and 2 mL of glacial acetic acid. This mixture was boiled at 100 °C for 30 min, followed by termination of reaction in an ice bath. The reaction mixture was extracted with 4 mL toluene, and the absorbance of the organic phase was subsequently read at 520 nm. The results were compared to a standard curve constructed using known amounts of proline. 

#### 4.4.6. Determination of MDA Content

For the determination of malondialdehyde (MDA) content, 200 mg of ground leaf tissue was homogenized with 2 mL of 10% trichloroacetic acid solution (TCA) and then centrifuged at 12,000× *g* for 10 min. Then, 1.5 mL of the supernatant was aspirated, and 1.5 mL of 0.6% thiobarbituric acid (TBA) in 10% TCA was added and heated in boiling water for 15 min and then quickly cooled in an ice bath and subsequently centrifuged at 12,000× *g* for 10 min. Absorbance was recorded in a spectrophotometer at 532 and 600 nm. The non-specific absorption at 600 nm was subtracted. The extinction coefficient of 155 mmol L^−1^ cm^−1^ for MDA was used.

### 4.5. Antioxidative Enzyme Activities Test

Superoxide dismutase (SOD, EC 1.15.1.1) and peroxidase (POD; EC 1.11.1.7) activities were tested according to methods described by Miao et al. (2010) [103]. An amount of 200 mg of ground leaf sample was homogenized in 20 mL of 50 mM ice-cold phosphate buffer (pH 7.8) containing 1% (*w*/*v*) polyvinylpyrrolidone. The homogenates were centrifuged at 4 °C for 10 min at 10,000× *g*. The resulting supernatant was used as a crude enzyme extract for the determination of the activities of antioxidant enzymes. SOD activity was determined spectrophotometrically at 560 nm per minute. One unit of SOD was defined as the amount of enzyme that inhibits the rate of nitroblue tetrazolium photoreduction by 50%. One unit of POD enzyme activity represents the amount of enzyme that increases by 0.01 of absorbance at 470 nm per minute. One unit of CAT (EC 1.11.1.6) was determined spectrophotometrically at 240 nm per minute as the amount of enzyme that decreases by 0.1 of absorbance [104]. 

### 4.6. RNA Extraction and Quantitative Real-Time PCR Analysis

Total RNA was isolated from stressed and unstressed samples using the RNeasy plant mini kit (Qiagen, Valencia, CA, USA) according to the manufacturer’s instructions. After digestion with an RNase-Free DNase (Ambion^®^ DNA-free^TM^DNase, Austin, TX, USA) to avoid possible genomic DNA contamination, first-strand cDNAs were synthesized from 2 µg RNA by using the Omniscript RT Kit (Qiagen, Hilden, Germany). Quantitative real-time PCR was conducted using the ABI PRISM 7900HT sequence detection system (Applied Biosystems, Foster City, CA, USA) and the SYBR Green PCR Master Mix. Relative fold changes were calculated based on the comparative Ct value using the 2^−ΔΔCt^ method. Actin was used as the internal reference. For each sample, measurements of three biological and three technical replicates were used. All gene-specific primers for qPCR are shown in Table 1.

### 4.7. Statistical Analysis Method

The data presented are expressed as average means ± SE of three independent biological and technical replicates. Data analysis was performed using Student’s *t* test. *p* values of <0.05 (*) and <0.01 (**) were considered statistically significant, and error bars indicate standard deviation.

## 5. Conclusions

In conclusion, the present study shows that *SlARF2AB-RNAi* transgenic tomato plants display significant amelioration in survival following salt and drought stresses, as seen in the increased contents of chlorophyll, soluble sugars, and proline, and in the scavenging excess ROS through the modulated antioxidant enzyme activities and the dynamic expression patterns of stress-related genes. These results suggest that *SlARF2* acts as a multifunctional regulatory protein in plant responses to abiotic stresses, providing new insights for the use of genetic editing in the incorporation of desirable traits including abiotic stress tolerance with yield potential and other agronomically valuable characteristics in horticultural crops.

## Figures and Tables

**Table 1:** Gene IDs and primer sequences used in this study.

Gene	Solyc ID	Forward Primer Sequence (5′–3′)	Reverse Primer Sequence (5′–3′)
*Sl-Actin*	Solyc03g078400	TGTCCCTATCTACGAGGGTTATGC	AGTTAAATCACGACCAGCAAGAT
		
		
*SlASR1*	Solyc04g071610.2.1	GGGACACCACCATCTCTTCTAAA	CCAAATATGGAAATTCCACGAATAT
		
*SlASR2*	Solyc04g071580.2.1	GACATTAATTTAAGAGAAGCAATACAATATGG	GGTGGAACAAATGGTGATGGT
		
*SlASR4*	Solyc04g071620.2.1	GGTAATGAGGAAGGTGGCTATGG	TGGTTCCACTATCATCATTCTCTTCA
*CI7*	Solyc04g082200.2.1	GGCAATTTCATCTGAGTTGTCTGA	CTATTTGATCGATGAAGTTTCTTTTCC
*SlSOD*	Solyc01g067740.2.1	TGAATTGGGGTTGAACCATT	GCAGGCACTGTAATCTGCAA
*SlCAT*	Solyc12g094620.1.1	TCCCAGTTAATGCTCCCAAG	CTCAGCAGGACGACAAGGAT
*SlPOD*	Solyc04g071900.2	CTTGCCCTAATGCTCTCACC	GCATCACAACCCTGAACAAA
*SlDREB1*	Solyc06g050520.1.1	GCAATGTCAGGAGCCGAATG	TCTTCTTGCCTGCCTGGTTT
*SlDREB2*	Solyc05g052410.1.1	GCAAGAGGACTTCCACTTCT	GCCATGTTGCCAATGCACCAA
*SlP5CS*	Solyc08g043170.2.1	TGCTGTAGGTGTTGGTCGTCA	TGCCATCAAGCTCAGTTTGTG
*SlERD15*	Solyc04g017690.2.1	AGGCATCAAGTCATCACTCTCTGGT	GAGGTAAATGTGAGTAAGAACCAACG

## Data Availability

All relevant data are within the paper.
